# Eco-Friendly Waterborne Polyurethane Coating Modified with Ethylenediamine-Functionalized Graphene Oxide for Enhanced Anticorrosion Performance

**DOI:** 10.3390/molecules29174163

**Published:** 2024-09-03

**Authors:** Mariel Amparo Fernandez Aramayo, Rafael Ferreira Fernandes, Matheus Santos Dias, Stella Bozzo, David Steinberg, Marcos Rocha Diniz da Silva, Camila Marchetti Maroneze, Cecilia de Carvalho Castro Silva

**Affiliations:** 1 Mackenzie School of Engineering, Mackenzie Presbyterian University, Consolação Street 930, São Paulo 01302-907, Brazil; reomldm@gmail.com (R.F.F.); theeus.santos@gmail.com (M.S.D.); stellabozzo98@gmail.com (S.B.); david.steinberg@mackenzie.br (D.S.); marcosrochadiniz@hotmail.com (M.R.D.d.S.); camila.maroneze@mackenzie.br (C.M.M.); 2 MackGraphe-Mackenzie Institute for Research in Graphene and Nanotechnologies, Mackenzie Presbyterian University, Consolação Street 930, São Paulo 01302-907, Brazil

**Keywords:** eco-friendly, waterborne polyurethane, coating, graphene oxide, low additive content, functionalization, anticorrosion, UV resistance, water resistance

## Abstract

This study explores the potential of graphene oxide (GO) as an additive in waterborne polyurethane (WPU) resins to create eco-friendly coatings with enhanced anticorrosive properties. Traditionally, WPU’s hydrophilic nature has limited its use in corrosion-resistant coatings. We investigate the impact of incorporating various GO concentrations (0.01, 0.1, and 1.3 wt%) and functionalizing GO with ethylenediamine (EDA) on the development of anticorrosive coatings for carbon steel. It was observed, by potentiodynamic polarization analysis in a 3.5% NaCl solution, that the low GO content in the WPU matrix significantly improved anticorrosion properties, with the 0.01 wt% GO-EDA formulation showing exceptional performance, high E_corr_ (−117.82 mV), low i_corr_ (3.70 × 10^−9^ A cm^−2^), and an inhibition corrosion efficiency (η) of 99.60%. Raman imaging mappings revealed that excessive GO content led to agglomeration, creating pathways for corrosive species. In UV/condensation tests, the 0.01 wt% GO-EDA coating exhibited the most promising results, with minimal corrosion products compared to pristine WPU. The large lateral dimensions of GO sheets and the cross-linking facilitated by EDA enhanced the interfacial properties and dispersion within the WPU matrix, resulting in superior barrier properties and anticorrosion performance. This advancement underscores the potential of GO-based coatings for environmentally friendly corrosion protection.

## 1. Introduction

Metal corrosion is a widespread issue that significantly affects human life in terms of economics, the environment, and health safety [1]. Anticorrosive coatings provide durability, cost-efficiency, and excellent protection for metal surfaces, mainly carbon steel, which represents the most used metal alloy in the world [2,3]. In this context, organic coatings are considered the most effective method for protecting carbon steel from corrosion, because they create a physical barrier against the aggressive environment [4,5,6,7]. The production of durable coatings with anticorrosive properties, while adhering to green chemistry principles (GCP), is currently an emerging challenge and a market demand [8,9,10,11]. Waterborne polyurethane (WPU) resins exemplify GCP, featuring low volatile organic compound content, which contributes to reducing emissions and air pollution [12,13]. WPU resins demonstrate excellent abrasion resistance. However, the hydrophilic segments in the polymer backbone, which contribute to the superior colloidal stability of WPU, also lead to some detrimental surface effects, including reduced water resistance, increased susceptibility to UV degradation, and vulnerability to corrosion. These factors collectively shorten the long-term application of WPU-based materials [14,15]. A great strategy to improve the long-term performance of WPU-based materials for anticorrosion applications is the development of nanocomposites [16,17]. The addition of nanoparticles in the polymer matrix really plays an important role in the improvement of the barrier properties and the water resistance [18,19]. In this scenario, graphene derivatives are appealing materials that have recently been applied to enhance the barrier properties of polymer-based materials [20,21,22].

Graphene and its derivatives, such as graphene oxide (GO) and reduced graphene oxide (rGO), have been widely utilized in the formulation of anticorrosive coatings due to their nano-barrier effect, which creates a labyrinth within the coatings, thereby prolonging the penetration path of corrosive species [13,23,24]. In particular, GO has attracted attention as an additive in coatings because of its impermeable properties, especially in water-based coatings [12,24,25]. This is due to its high dispersibility in water, which is attributed to its hydrophilic nature and the presence of oxygen-containing functional groups (hydroxyl, carboxyl, and epoxy groups) on its surface and edges [26]. However, achieving uniform GO dispersion within the polymeric matrix remains a common challenge highlighted in the literature [27]. This issue could potentially compromise the anticorrosive performance of the coatings since a poorly dispersed GO on the coating results in the creation of paths within the coating due to the agglomeration of the sheets [27,28,29]. Functionalization of GO is a promising strategy for promoting the uniform dispersibility of GO sheets in the coating polymer matrix [9,30]. Ning et al. functionalized GO with dodecylbenzenesulfonic acid, phosphoric acid, and polyaniline. This composite demonstrated improved dispersion and compatibility in WPU, thereby delaying the time for corrosion species to access the metal interface [31]. Wen et al. demonstrated the improvement in anticorrosion properties in WPU by incorporating 0.3 wt% GO covalently functionalized with isophorone diisocyanate [30]. Similarly, Cui et al. achieved comparable results by incorporating 0.2 wt% GO functionalized with polycarbodiimide into the WPU matrix [9]. Li et al. also described superior anticorrosion properties by incorporating 0.4 wt% rGO functionalized with titanate coupling agents into WPU [32]. A simple and cost-effective approach that has attracted attention involves using ethylenediamine (EDA) to functionalize graphene oxide (GO), because of its ability to manipulate the interlayer spacing between GO sheets [33]. Maslekar et al. determined the reactivity of EDA toward the main oxygen-containing functional groups of GO (epoxy, hydroxyl, and carboxylic acid) in lithium-ion batteries. Their findings highlighted a significant impact on properties, such as colloidal stability. For anticorrosive coatings, this enhanced colloidal stability is particularly valuable as it improves the uniform dispersion of GO flakes within the resin, thereby enhancing the effectiveness of the anticorrosive coating [33].

Most reports in the literature have achieved enhanced anticorrosion properties of WPU coatings by incorporating a high content (at least 0.2 wt%) of functionalized GO as an additive [8,9,16,18,30]. Song et al. reported that adding GO to WPU improved the water contact angle compared to pure WPU films, indicating enhanced hydrophobic properties. Nevertheless, they also noted that incorporating more than 0.5 wt% of GO decreased the tensile strength of the films [8]. Thus, it remains essential to balance effective functionalization to prevent agglomeration with determining the optimal concentration of GO within the polymeric matrix. However, a common limitation in many studies is the lack of long-term accelerated testing, which is crucial to fully evaluate the performance and durability of these coatings.

Aiming to improve GCP principles by reducing the amount of nanomaterial used, we present an investigation into the effect of incorporating different concentrations of GO (1.3, 0.1, and 0.01 wt%) in WPU resins and the impact of functionalizing a low content (0.01 wt%) of GO sheets with ethylenediamine (EDA) as an additive to develop anticorrosive coatings for carbon steel surfaces. The functionalization method using EDA, a commercially available single-component reagent, is not only straightforward and cost-effective but also highly efficient. It requires fewer materials and less time than the more complex methods reported in the literature [18,31,34,35,36,37,38]. Potentiodynamic polarization analysis in a 3.5% NaCl solution established a strong correlation between the low GO content in the WPU resin matrix and higher anticorrosion properties, especially for the 0.01 wt% GO-EDA. The large lateral size of the GO sheets, combined with the cross-linking between GO and WPU promoted by EDA, improved the interfacial properties between the GO and WPU polymer matrix. This resulted in better barrier properties and a less hydrophilic surface, consequently leading to a higher anticorrosion performance of the developed eco-friendly coating.

## 2. Results and Discussion

### 2.1. Characterization of GO

**Figure 1:**
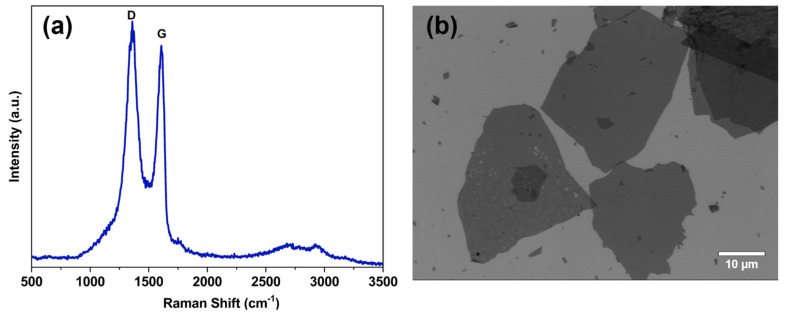
(**a**) Representative Raman spectrum of GO sheets used to prepare the nanocomposites with WPU, displaying the typical D and G bands. (**b**) SEM micrograph of GO sheets on a Si substrate.

The successful oxidation of the graphite and the achievement of GO sheets were confirmed by the well-defined Raman spectrum of GO presented in Figure 1a and UV-Vis spectra of GO dispersion (Figure S1). In the Raman spectrum, Figure 1a, the D band at ~1350 cm^−1^ is related to the defects in GO structures (sp^3^ carbons from the oxygen functional groups); the G band at ∼1610 cm^−1^ is attributed to the sp^2^ carbons (refers to the stretching of the C–C bonds), where its intensity and position indicate the degree of graphitization; and the absence of the 2D band (usually at ~2700 cm^−1^) is related with the high structural disorder degree in the two-dimensional plane of GO [39,40]. Figure S1 shows the characteristic UV-Vis spectra for GO dispersion, exhibiting two bands at approximately 230 and 300 nm, respectively, attributed to π → π* transitions, associated with C–C bonds in aromatic species, and n → π* transitions, related to C–O bonds, resulting from oxidation processes [41,42,43].

The morphology and lateral size of the GO sheets were examined using scanning electron microscopy (SEM), as shown in Figure 1b and Figure S2. This analysis underscores graphite’s effective oxidation and exfoliation, resulting in GO sheets distinguished by their transparency and large lateral dimensions exceeding 25 μm. This transparency indicates the presence of only a few layers of GO [44,45].

### 2.2. Dispersion of GO in the WPU Matrix

**Figure 2:**
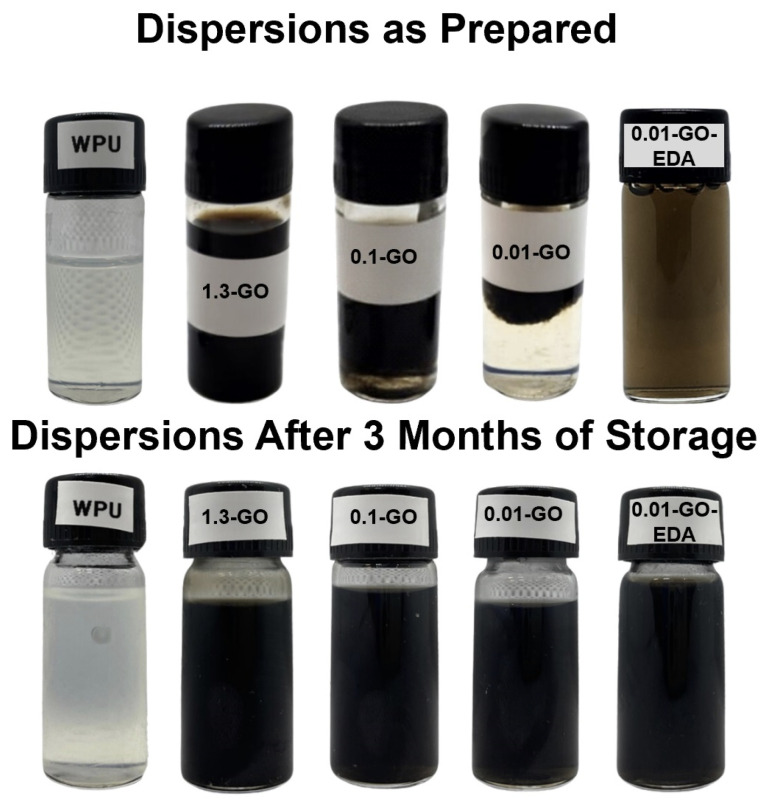
Photographic images of WPU resin and dispersions of GO in WPU resin at various concentrations after preparation and 3 months of storage.

To evaluate the stability of GO in the WPU resins, the dispersions were stored for three months. Figure 2 shows photographic images of the WPU resin and GO dispersions in WPU at various concentrations, both immediately after preparation and after three months of storage. After these months, a characteristic black color was observed in the dispersions, which can be attributed to the mild partial reduction of the GO to rGO, as also observed by Otsuka et al. for GO aqueous colloidal suspension after seven days [46]. All samples remained stable except for 0.01-GO and 0.1-GO, likely due to their low GO concentrations. However, mechanical agitation restored 0.1-GO stability. The functionalization with EDA was performed to promote the better dispersibility of GO sheets on the WPU matrix for the 0.01-GO sample. The EDA can interact with GO by hydrogen bonding and/or as a cross-linking agent between the GO sheets since the two terminal amino groups (-NH_2_) can covalently bind to the carboxyl groups of GO sheets, promoting a higher dispersion of GO sheets into the WPU matrix [47]. This potentially improves the nanocomposite’s barrier properties [47,48]. Additionally, since EDA acts as a hardener for the polyurethane resin, it may enhance the mechanical resistance of the material. However, it is crucial to use the appropriate amount (low content) of this chain extender to achieve optimal results, avoiding the self-polymerization process between the EDA and the WPU [49,50].

**Figure 3:**
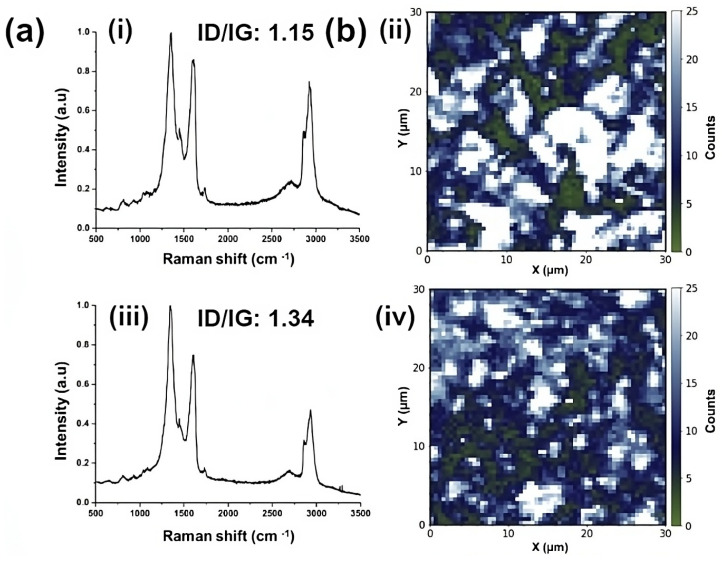

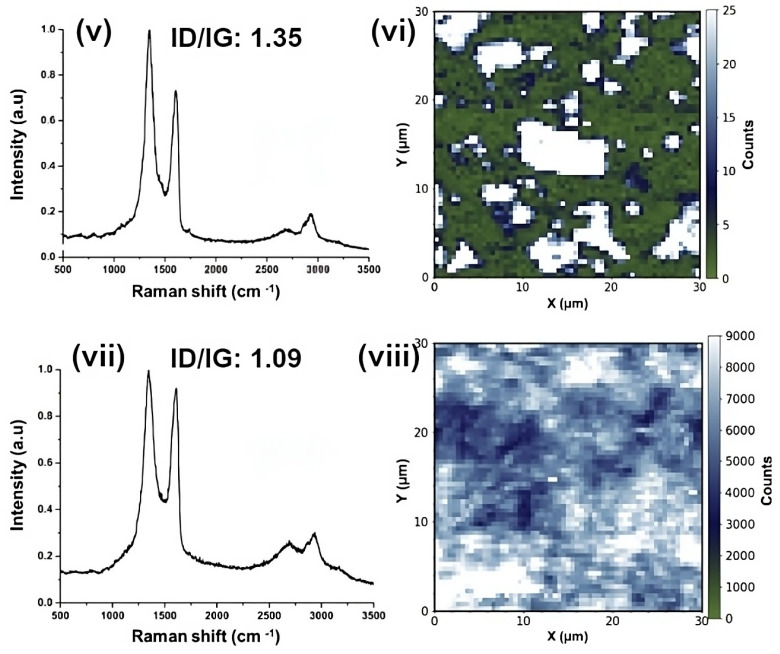
(**a**) Representative Raman spectrum, ID/IG intensity ratio, and corresponding (**b**) Raman imaging mapping, performed using the G band intensity as the reference for the samples, for (**i**,**ii**) 0.01-GO; (**iii**,**iv**) 0.01-GO-EDA; (**v**,**vi**) 0.1-GO; and (**vii**,**viii**) 1.3-GO.

Raman analysis and 2D imaging mappings of a section of the coating were performed to confirm the incorporation and dispersibility of GO in the WPU resin as a function of GO content. Figure 3(a-i) shows the representative Raman spectrum for the 0.01-GO sample. It is possible to identify the typical D (1356 cm^−1^) and G (1607 cm^−1^) band for GO sheets. The ID/IG intensity ratio measures the disorder degree and the average size of the sp^2^ domains in graphite materials [39,40]. For the 0.010-GO sample, it was found to be 1.15. For the identification of the WPU, the presence of the Raman bands was detected with a maximum at 2932 cm^−1^ and 2863 cm^−1^, which can be attributed to asymmetric and symmetric C-H stretching vibration of CH_2_ groups, and the Raman band at 1732 cm^−1^ can be related to the C=O stretching vibration modes of the ester group of the polyurethanes [51]. Figure 3(b-ii) shows the respective Raman imaging mapping for the 0.01-GO sample, which was constructed based on the intensity of the G band. It is possible to observe some regions of high intensity in the G band (white color), which may be related to the non-uniform dispersibility of the GO sheets in the WPU in this concentration, as also observed in Figure 2. Supplementary Video S1, associated with the real-time Raman mapping of the 0.01-GO sample, makes it possible to observe some dark areas in the mapping, where is not possible to observe the D and G band of the GO Raman spectrum with enough intensity, corroborating with the low uniform distribution of the GO sheets in the WPU matrix. Figure 3(a-iii,b-iv) shows the effect of functionalizing the 0.01-GO sample with EDA. The Raman spectrum of the 0.01-GO-EDA sample exhibits the same Raman features as the 0.01-GO sample. However, after functionalization with EDA, the ID/IG intensity ratio increased from 1.15 to 1.34. This indicates a decrease in the size of the in-plane sp^2^ crystalline domains, which may be related to the chemical functionalization and partial reduction in GO through its reaction with EDA [52]. The carboxyl and epoxy groups of GO can react with the amine groups of the EDA molecule, partially restoring the sp^2^ crystalline domains but with smaller sizes [47]. This increases the intensity of the D band due to border defects (edges). The improvement in the homogeneous dispersibility of GO sheets after functionalization with EDA is further evidenced by the Raman imaging mapping (Figure 3(b-iv)) and Supplementary Video S2. A significant decrease in the area of GO agglomeration (white color) can be observed. Additionally, the presence of GO sheets is visible throughout the entire extent of the samples. For the samples with higher GO content in the WPU matrix, 0.1-GO and 1.3-GO, a similar Raman spectrum was observed (Figure 3(a-v,vii)). The D and G bands are present; however, the Raman feature related to the C=O stretching vibration modes (1732 cm^−1^) of the ester group in the polyurethanes was not observable. Besides that, the intensity of the asymmetric and symmetric C-H stretching vibrations of CH₂ groups significantly decreased, likely due to the high content of GO sheets in the polymer matrix, which dominate the Raman signature signal. An ID/IG intensity ratio of 1.35 and 1.09 was found for the 0.1-GO and 1.3-GO samples, respectively. The Raman imaging mapping (Figure 3(b-vi,viii)) and Supplementary Videos S3 and S4 clearly show the agglomeration process that occurs with the GO sheets in the WPU matrix as the GO content increases. In both cases, regions with a high agglomeration of GO sheets (white areas in the Raman imaging mapping—Figure 3(b-vi,viii)) are evident. In the 0.1-GO samples, several areas without GO sheets (black areas in the Raman imaging mapping—Supplementary Video S3) were also observed.

### 2.3. Evaluation of Chemical Interaction of GO with WPU Resin

**Figure 4:**
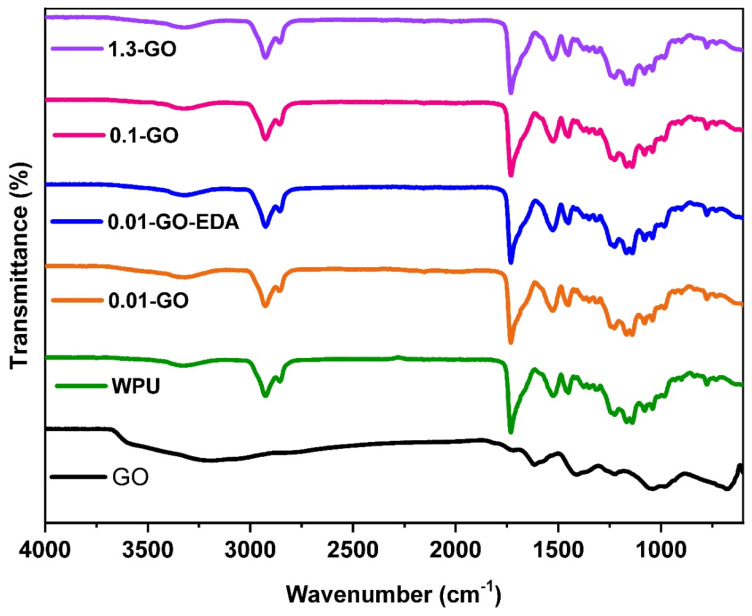
FTIR spectra of GO, WPU resin, and all concentrations of GO incorporated into the WPU resin.

Figure 4 shows the FTIR spectra of GO, WPU, and the coated steel samples of 1.3-GO, 0.1-GO, 0.01-GO, and 0.01-GO-EDA. The FTIR spectrum of the GO sample exhibits the characteristic features of graphene oxide. Bands in 1720, 1620, and 1411 cm^−1^ correspond to the vibrational modes of carbonyl groups (C=O), alkene groups (C=C), and hydroxyl groups (–OH), respectively [53,54,55,56]. Additionally, the bands in 1045 and 979 cm^−1^ are attributed to the epoxide groups [53,54,55,56].

The spectrum of WPU (green curve) displays the functional groups attributed to polyurethane resin. The band at 3300 cm^−1^ is attributed to the N-H stretching vibration of the urethane bond of PU. The two bands at 2930 cm^−1^ and 2850 cm^−1^ correspond to the C-H stretching vibrations of urethane bonds. The absorption band at 1740 cm^−1^ is attributed to the C=O stretching vibration of the urethane group [8,9,14,15,57]. The FTIR spectra of WPU and WPU containing the different concentrations of GO are similar because the content of GO incorporated into the WPU matrix is minimal in relation to the presence of the WPU, causing the GO bands to overlap with the more intense bands of the WPU resin. The same behavior is observed for the 0.01 GO-WPU sample functionalized with EDA. It was not possible to verify the presence of the C–N stretching (related to the covalent bond between the oxygen functional groups of GO, such as epoxy and carboxyl, and the amino groups of EDA) [47] through FTIR analysis, as this feature was already obscured by the C–N stretching of WPU (~1453 cm^−1^) [58]. Similarly, the –NH bands of the primary (3300 cm^−1^) and secondary amines (1570 cm^−1^) in EDA [47] were masked by the –NH stretching (3500–3300 cm^−1^) and –NH bending (1583–1486 cm^−1^) in the WPU [9].

### 2.4. Hydrophobicity Analysis

**Figure 5:**
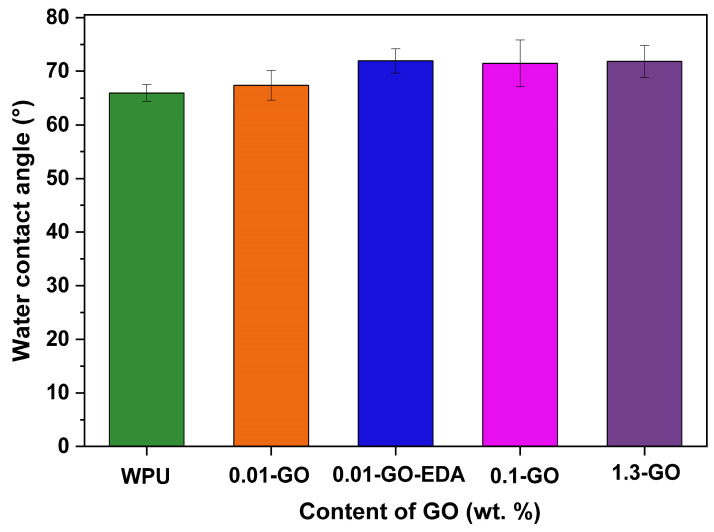
The water contact angle of WPU resin is a function of incorporating different GO and EDA-functionalized GO contents applied to the carbon steel substrates.

Figure 5 shows the results of the static contact angle measurements used to examine the hydrophobicity of GO-WPU coatings applied on carbon steel sheets. The waterborne polyurethane resin without GO already exhibits an intrinsic low hydrophobic character. Nevertheless, the incorporation of GO increased the contact angle, hence increasing the hydrophobicity of the coating. This improvement is primarily due to the graphitic structure of GO, which produces a higher hydrophobic surface, in comparison with the pristine WPU surface, that resists water spreading [24,59,60]. The increase in GO content from 0.01 to 1.3 wt%. increased the contact angle of the water on the WPU films from 67.35° to 71.82°, compared to 65.92° for the neat WPU. Song et al. found that the maximum water contact angle reached for 1.0 wt% GO was 55.38°, with flakes measuring 105.42 nm on average. In our study, the GO flakes possess an average size of around 25 µm, which may contribute to the enhanced contact angle due to improved hierarchical surface structures and roughness in the coating [21,24]. Additionally, Song et al. reported that an optimal GO content in WPU up to 0.5 wt% could prevent GO agglomeration in the resin [8]. In the present work, the coatings with the lowest concentration studied (0.01 wt% GO) and functionalized with EDA (sample 0.01-GO-EDA, in Figure 5) demonstrated the highest contact angle (71.92°). This can be attributed to the chemical functionalization and partial reduction of GO through its reaction with EDA. The carboxyl and epoxy groups of GO react with the amine groups of the EDA molecule, promoting the mild reduction in GO and improving the hydrophobicity of the surface [57]. This enhancement in hydrophobicity is a critical aspect of achieving high anticorrosion properties [8,22]. Reducing water permeation through WPU represents one of the biggest challenges in using this polymer to develop coatings with barrier properties [16]. In this work, we achieved promising results using a low content of GO (only 0.01 wt%) due to the combination of the sizeable lateral size of the sheets and the functionalization with EDA.

### 2.5. Evaluation of Anticorrosion Performance

**Figure 6:**
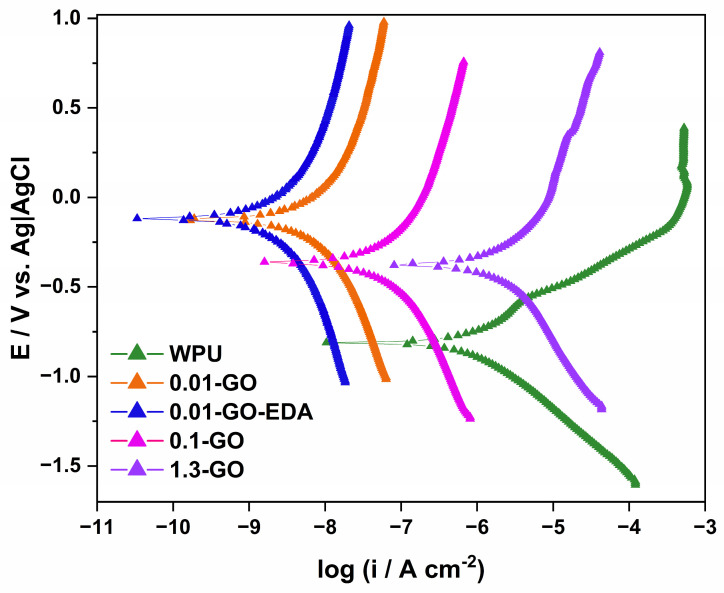
Potentiodynamic polarization curves of WPU resin and various concentrations of GO incorporated into the WPU resin in 3.5% NaCl aqueous solution.

Potentiodynamic polarization curves were measured for all studied conditions to evaluate the effect of GO incorporation on the anticorrosive performance of coated carbon steel in a 3.5% NaCl solution. The results are illustrated in Figure 6.

From the corrosion parameters, such as the corrosion current density (i_corr_) and the corrosion potential (E_corr_) extracted from Figure 6, it was possible to calculate the corrosion rate and the coating inhibition efficiency (η), which is determined using Equation (1) [61,62]:

(1) $$\eta =1-\frac{{i^{GO}}_{corr}}{i_{corr}}$$

where ${i^{GO}}_{corr}$ is the corrosion current density for the WPU-coated samples containing graphene oxide, and $i_{corr}$ is the corrosion current density for the coated samples with pristine WPU. All these corrosion parameters are summarized in Table 1.

The Figure 6 shows that the corrosion potential of the samples containing GO shifted to a more noble potential than that of the sample covered exclusively with WPU resin. This indicates a decrease in their susceptibility to corrosion (see Table 1) [63,64]. As observed from the contact angle results, the incorporation of GO increased the contact angle of the surface, improving the hydrophobicity of the WPU-modified coatings and, therefore, the water barrier permeation. However, it also altered the rate of oxidation–reduction reactions occurring on the coating surface, as indicated by the electrochemical test. When comparing the pristine WPU resin to WPU resin containing GO, the latter shows a two-order magnitude decrease in corrosion current density. This decrease can be attributed to the addition of GO and functionalized GO in the polymeric matrix, which improves the barrier properties of the coatings [12,25]. Among all samples studied, the 0.01-GO-EDA sample exhibited the best anticorrosion behavior, with the highest E_corr_ value (−117.82 mV) and the lowest i_corr_ value (3.70 × 10^−9^ A cm^−2^). Generally, lower I_corr_ and higher E_corr_ indicate more protective properties against corrosion [63]. The corrosion rate and inhibition efficiency (η) data show that the 0.01-GO and 0.01-GO-EDA samples exhibit lower corrosion rates (1.05 × 10^−^⁴ mm/year and 4.15 × 10^−^⁵ mm/year, respectively) and higher inhibition efficiencies (99.00% and 99.60%), indicating enhanced corrosion protection compared to the 1.3-GO and 0.1-GO samples. The corrosion rate of pristine WPU is lower than that of the 1.3-GO sample, suggesting that a high GO content accelerates corrosion, thereby compromising the protection offered by the pristine WPU.

**Figure 7:**
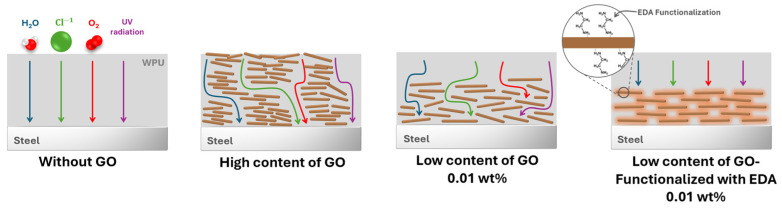
Schematic illustration depicting the effect of different contents of GO and EDA-functionalized GO on the barrier protection of the coating.

The functionalization of 0.01-GO-WPU with EDA enhances performance compared to the 0.01 wt% GO sample, which, although still effective, exhibits a slightly lower E_corr_ of −126.30 mV and a higher i_corr_ of 9.34 × 10^−^⁹ A cm^−2^. This demonstrates that the functionalization of GO with EDA further improves the barrier properties of the coating. In contrast, the 0.1% GO sample shows a higher i_corr_ (1.02 × 10^−7^ A cm^−2^) than the 0.01 wt% GO samples. This indicates that increased GO content does not necessarily improve anticorrosion behavior and may lead to more agglomeration than the 0.01 wt% GO samples. This suggests that increasing GO content does not necessarily improve anticorrosion performance and may lead to agglomeration issues of GO flakes. The 1.3-GO sample, which shows an even higher corrosion current density than both the 0.1-GO sample and pristine WPU resin, further confirms that excessive GO content can compromise the coating’s barrier properties as observed in Figure 6 and Raman imaging mappings (Figure 3(b-vi,viii)) and videos (Supplementary Videos S3 and S4). Heiba et al. reported that incorporating a low content of GO can enhance corrosion protection due to better uniform distribution of GO sheets in the polymer matrix [63]. Chen et al. reported that the use of high-content of nanoparticles tended to agglomerate when added to a polymeric matrix [65]. In our study, the GO flakes measured approximately 25 µm in size, which can improve coating protection by establishing a nano-barrier effect, which creates a labyrinth within the coatings, thereby prolonging the penetration path of corrosive species [4,22,27]. However, inadequate dispersion of GO sheets can lead to the formation of preferential pathways within the coating, allowing for aggressive species to easily reach the metal, thereby reducing its barrier protection, as illustrated in Figure 7.

Our findings regarding the anticorrosion properties of the developed coating 0.01-GO-EDA based on WPU are much more promising than those of several reports in the literature, even in relation to those exploring the use of epoxy resins to develop the GO coatings. Table 2 summarizes recent works reported in the literature on developing coatings with anticorrosion properties based on the incorporation of graphene derivatives as an additive. The respective results regarding corrosion potential (E_corr_) and corrosion current (i_corr_) are presented.

As demonstrated in Figure 6 and Table 1, the samples 0.01-GO and 0.01-GO-EDA exhibited the lowest corrosion current. Therefore, these concentrations and the pristine WPU resin were selected for the durability investigation test.

**Figure 8:**
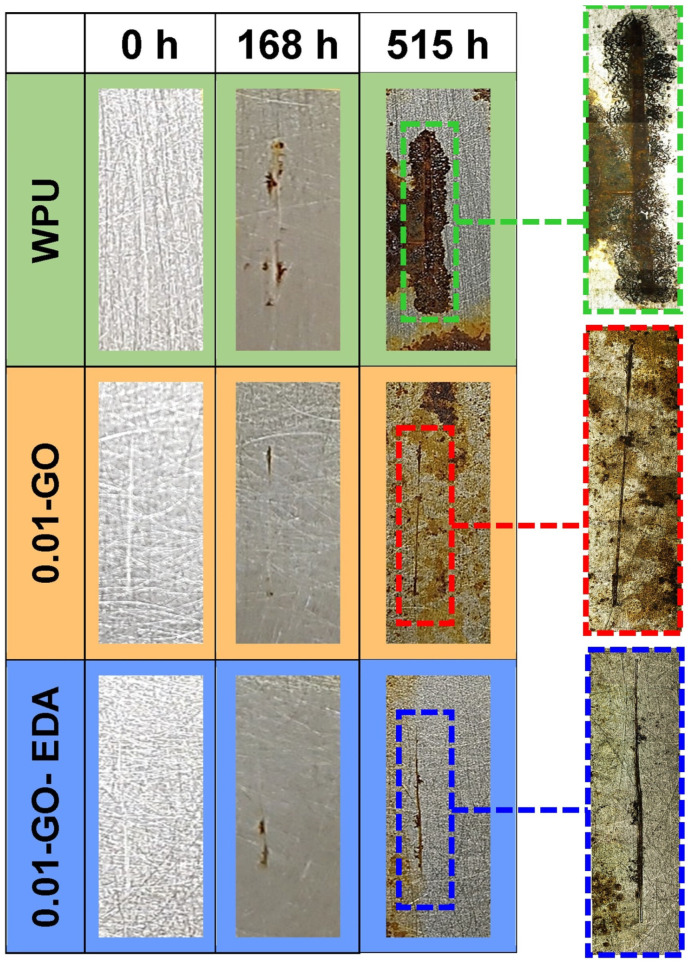
Photographic images of coated samples with artificial defects exposed to UV/condensation tests after 168 and 515 h, along with their respective optical microscopic images of the defect sites.

Figure 8 shows the surface morphology of the coated samples with artificial defects after 168 h and 515 h of exposure to a UV/condensation test. As observed in the images, prior to the accelerated aging test, the coated samples showed the characteristic brightness of WPU resin. Duong et al. reported that after 216 h of UV/condensation testing, polyurethane coatings (solvent-based) without GO degraded with loss in gloss, suggesting UV radiation-induced degradation. However, samples containing 0.01 wt% GO exhibited less gloss degradation. This can be attributed to the properties of GO in absorbing UV light, increasing the UV durability of the polyurethane coating [12]. In our study, after 515 h of exposure, we observed that the pristine WPU resin lost its brightness, while the 0.01-GO-EDA formulation exhibited less degradation, highlighting the beneficial role of the functionalized EDA-GO even at low concentrations. Furthermore, the involvement of EDA in creating a more compact cross-linking matrix and hydrophobic surface, contributes to a slower degradation of the film than using pristine WPU resin [49,50]. The evaluation of brightness in the 0.01-GO sample was complex because of the presence of corrosion products developed on its surface, likely caused by GO agglomeration that created preferential pathways through the coating to the metal, thereby triggering premature corrosion. In contrast, the 0.01-GO-EDA sample revealed a small quantity of formation of cracks in the resin after exposure, indicating that aggressive species did not easily penetrate the coating. This finding confirms that the functionalization of GO with EDA not only enhanced the formation of a dense three-dimensional network but also improved the dispersion of GO in the coating and the hydrophobicity of the coating surface, as illustrated in the schematic representation of Figure 7.

After 168 h of exposure, corrosion products were observed at the artificial defect sites in all samples, with a notable increase in quantity after 515 h. Although corrosion progressed in all studied conditions, the extent of propagation from the defect sites differed among samples. The pure resin showed the most severe corrosive attack, as evidenced by the most corrosion products at the defect site. The low anticorrosion performance of the pure resin coating after the aging test can be attributed to surface deterioration caused by UV exposure, which compromised its barrier properties. In contrast, the 0.01-GO sample exhibited fewer corrosion products, and the 0.01-GO-EDA sample even less, indicating that the addition of GO and functionalized GO increased the corrosion resistance of the coating after UV/condensation aging. According to ISO 4628-8 standards, the corrosion around the scribe can be classified as grade 5 (severe) for the WPU-coated sample, and grade 2 (slight) for both the 0.01-GO and 0.01-GO-EDA samples [66]. This analysis indicates that the UV/condensation exposure induced physical changes in the coatings due to the combined effects of UV radiation, temperature, and condensation. Catastrophic macroscopic failures start from microscopic physical changes in the coating, often triggered by preceding chemical alterations [67].

## 3. Materials and Methods

### 3.1. Materials

Graphite crystals with lateral sizes of 9 mm to 12 mm from Nacional de Grafite (Itapecerica, Brazil) were used in the preparation of GO along with sulfuric acid (H_2_SO_4_) P.A. (98.0% purity), hydrochloric acid (HCl) P.A. (37.0% purity), potassium permanganate (KMnO_4_) PA (99.0% of purity), and hydrogen peroxide (H_2_O_2_), 30 volumes, all of these chemicals from Synth (São Paulo, Brazil). The Ultra-pure water from the Milli-Q commercial system by Millipore Corporation (Burlington, MA, USA) with a resistivity of 18.2 MΩ.cm and TOC 2 ppb. The sodium nitrate (NaNO_3_) PA (99.9% purity) from Merck (Darmstadt, Germany). The EDA (MW = 60.1 g mol^–1^) (99% purity) was used to functionalize GO-WPU from Merck (Darmstadt, Germany).

AISI 1070 carbon steel sheets with dimensions of 10 mm × 30 mm × 2 mm were used as working electrodes. The type of carbon steel used was determined by energy-dispersive X-ray spectroscopy (EDS) analysis (field-emission scanning electron microscopy (SEM) model JSM-7800—Oxford Aztec, INCA) from JEOL Ltd. (Tokyo, Japan), presented in Supplementary Materials, Figure S3. Renner Sayerlack Company (Cajamar, Brazil) provided the coating, a single-component waterborne aliphatic polyurethane resin with a pH of 8.5, density of 1.05 g cm^−3^, and viscosity of 150cP. The manufacturer specifies the volume solids percentage of the mixture to be 36 ± 2%.

The electrolyte utilized in the potentiodynamic polarization tests was 3.5% NaCl solution from Synth (São Paulo, Brazil).

### 3.2. Methods

#### 3.2.1. Preparation of Graphene Oxide

Graphene oxide (GO) was synthesized according to the well-developed Hummer’s modified method [68,69] with a total oxidation time of three days and purified according to the process described by Rocha [70]. In a 100 mL round-bottom flask, 0.5 g of graphite flakes and 16.9 mL of H_2_SO_4_ were added. This mixture was stirred for 30 min using a magnetic stirrer in an ice bath. After 30 min, 0.38 g of NaNO_3_ was added. The solution was agitated for 5 min to ensure proper incorporation, and then 2.25 g of KMnO_4_ was gradually added over 1 h while stirring and using an ice bath. After the KMnO_4_ addition, the system was stirred for 24 h before resting for 72 h at room temperature to oxidize the material. After resting, 50 mL of H_2_SO_4_ at a concentration of 0.06 mol L^−1^ was gradually added over 1 h, with continued stirring and ice bath. Subsequently, 1.5 mL of 30% H_2_O_2_ was slowly added while maintaining stirring in an ice bath. The prepared GO dispersion was washed three times with a 10% hydrochloric acid aqueous solution to finish the process. The GO dispersion was purified using dialysis bags (porosity of 12 kDa, from Sigma Aldrich (San Luis, MO, USA)) in distilled water. The ultrapure water was changed until a pH of 5.5 was achieved. The GO dispersion was concentrated using a rotary evaporator (Q344M from QUIMIS Aparelhos Científicos, São Paulo, Brazil) to reach a concentration of 4.97 mg mL^−1^.

#### 3.2.2. Preparation of GO Waterborne Polyurethane Composite Coatings

**Figure 9:**
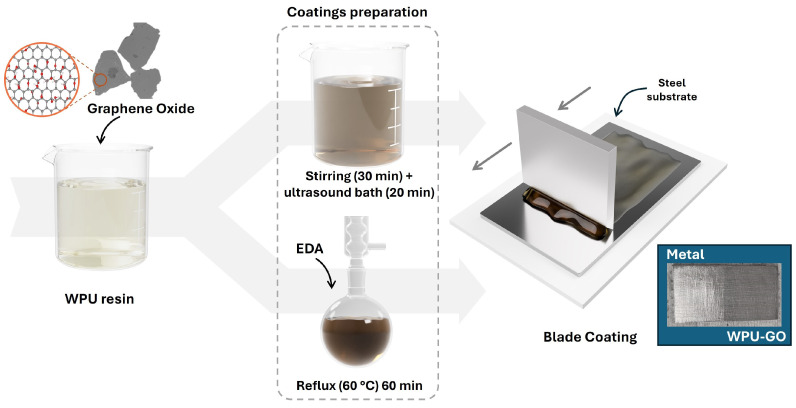
Schematic illustration of the preparation and application of GO-WPU and GO-EDA-WPU coatings on carbon steel sheets. Photograph image of a representative WPU-GO coating applied by blade coating on the carbon steel sheet showing the uniformity of the deposited film.

The graphene oxide/waterborne polyurethane (GO/WPU) composite coating was prepared by uniformly dispersing GO in the resin. The procedure can be observed in Figure 9. After adding the GO to the WPU resin, it was subjected to magnetic stirring for 30 min, followed by sonication on an ultrasound bath for 20 min at 37 kHz.

Based on the quantity of the solids in the pure resin, the GO was incorporated at concentrations of 1.3 wt%, 0.1 wt%, 0.01 wt%, and 0.01 wt% functionalized with EDA. These conditions are designated as 1.3-GO, 0.1-GO, 0.01-GO, and 0.01-GO-EDA, respectively. Control samples, referred to as WPU, were also evaluated, representing the waterborne polyurethane resin without GO.

The functionalization of 0.01 wt% GO with EDA was also evaluated. Inspired by the work of Jang et al. [47], who prepared a cross-linked GO membrane by functionalization with EDA, 30 μL (0.45 mmol) of EDA were added to 10 mL of WPU resin containing 0.01 wt% GO under constant stirring at 1300 rpm at room temperature for five minutes. Subsequently, the mixture was subjected to vigorous magnetic stirring and heating at 60 °C (silicone bath) in a reflux system for sixty minutes. After this period, the 0.01-GO-EDA sample was ready for use.

The carbon steel sheets were evenly sanded with 100-grit sandpaper. The coating film was then applied to the treated sheets using a Blade Coater, model BCC-02-V3 from Autocoat (Campinas, Brazil). This device uses a knife-shaped blade set at a 30-degree angle, with a height of 100 µm relative to the substrate and a deposition speed of 20 mm s^−1^ at an ambient temperature of 25 °C. The coatings were cured at room temperature for 24 h to obtain the final coated samples. After drying, the final single coating layers achieved an average thickness of 44.3 ± 3.8 μm, as measured by a hand-held electronic gauge, model 456C, from Elcometer (Manchester, UK).

#### 3.2.3. Characterization

The morphology of the GO flakes was characterized by field-emission scanning electron microscopy (SEM) (Jeol, model JSM-7800), operated at 0.3 keV of accelerating voltage. The GO samples, with concentrations of 0.001 mg mL^−1^, were prepared by drop casting on Si substrates. The type of carbon steel used was also evaluated by SEM images and determined by energy-dispersive X-ray spectroscopy (EDS) analysis (Jeol, model JSM-7800—Oxford Aztec, INCA).

The FTIR technique was employed to chemically characterize the GO-WPU composite coatings. All measurements were conducted in a Shimadzu (Tokyo, Japan) IRAffinity 1S FTIR spectrometer using attenuated total reflection (ATR). The spectra were obtained in the region from 600 to 4000 cm^−1^.

The chemical and spatial characterization of GO in the WPU was achieved utilizing a Raman spectrometer from Witec, Oxford Instruments Group (High Wycombe, UK), Aplha 300R model, coupled to a confocal optical microscope, with a laser set at 532 nm, 0.5 mW of power, 600 gr mm^−1^ grating, and BLZ of 500 nm. The spectra were obtained with an integration time of 10 s and 10 accumulations, utilizing lenses with 10× and 50× magnification. The Raman mappings were performed to identify the effective dispersion of the GO in the resin. The microscopic image of the resin film containing the GO sheets was initially obtained. Then, using the conditions of 2 s of integration, 15 accumulations, and lenses with 10× and 50× magnification, areas of 30 × 30 µm were analyzed to identify the D and G bands, as well as the central band of the resin. The obtained spectra were analyzed using Python language by Google Colaboratory software (https://colab.research.google.com/, accessed on 11 May 2024) software, which attributed different colors to their composition and generated images of the analyzed region. The Raman spectra were obtained in the range of 500 to 3500 cm^−1^. The successful achievement of GO dispersion was also evaluated by UV-Vis-NIR spectroscopy, which was performed on a Shimadzu spectrophotometer, model UV-3600, with a scanning range of 200 to 800 nm and an optical path length of 1.00 cm, controlled by UV-Probe software, version 2.62.

The wettability of coated carbon steel sheets was evaluated by static contact angle measurements utilizing the sessile drop method. An average of twenty measurements were taken to report the wettability. For each measurement, a 1 µL droplet of deionized water was deposited on the coated sample surface. The equipment employed was a Drop shape analyzer (DSA100) from KRUSS (Hamburg, Germany), operated with ADVANCE software, 2017 version.

The optical microscopy images of the carbon steel samples coated with the WPU, WPU-GO and WPU-GO-EDA after the weathering test were achieved by an optical microscope from Olympus, model BX51M (Tokyo, Japan) with a magnification of 5×.

#### 3.2.4. Electrochemical Characterization and UV/Condensation Exposure Test

**Figure 10:**
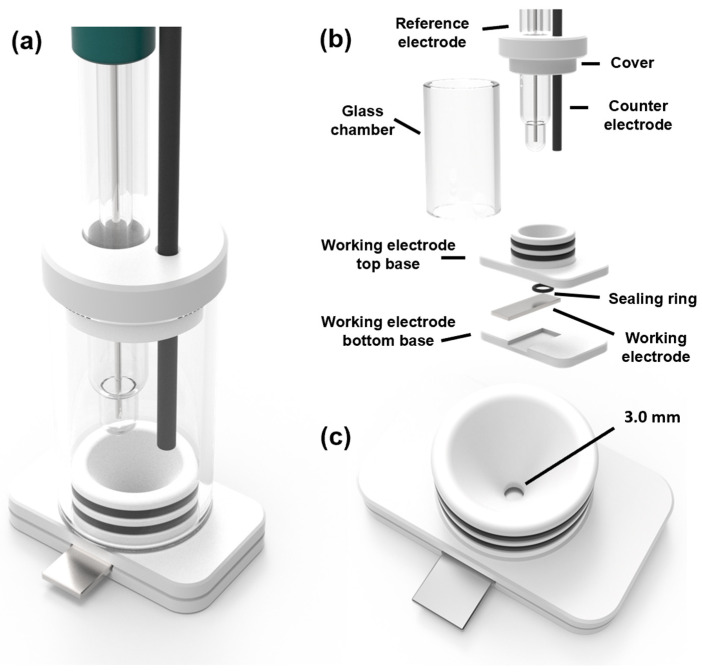
(**a**) Schematic diagram of a three-electrode cell used for electrochemical testing, (**b**) components of the cell, and (**c**) description of the cell base showing the test area for the samples.

The electrochemical test workstation used to assess the protective properties of the coatings by potentiodynamic polarization (PDP) analysis was the Autolab SS101, manufactured by Methrom (Herisau, Switzerland). Figure 10 displays the three-electrode system utilized, showing the components of it. The coated samples served as the working electrode with an exposed area of 7.1 mm^2^. A graphite rod electrode was utilized as the counter electrode, and an Ag/AgCl electrode as the reference electrode. The PDP curves were conducted in the potential range of 1 V on either side of Ecorr and at a scan rate of 0.01 V/s. The test corrosive solution was 3.5 wt% NaCl. All conditions were repeated three times under environmental conditions to confirm the reproducibility of the results.

The PDP analysis identified the two most effective concentrations of GO in WPU, which exhibited the lowest corrosion current, and these were compared with the pure WPU resin. These three conditions were then subjected to a weathering test that was conducted according to ASTM standard G51 [71]. First, an artificial defect, approximately 10 mm in length, was created on the surface of each coated carbon steel sample using a cutter, exposing the underlying steel. The samples were then placed in a UV/condensation chamber (QUV/spray with Solar Eye Irradiance Control from QLab) designed to simulate accelerated weathering conditions. During the test, the samples were cyclically exposed to UV-B radiation (310 nm) at 0.71 W/m^2^ for 4 h at 60 °C, followed by 4 h of humidity condensation at 50 °C. The coated carbon steel panels (10 × 30 × 2 mm) were subjected to a total of 515 h of exposure. Samples were removed at intervals of 168 h and 515 h to assess their resistance to weathering. After the test, photographic and optical microscopic images of the coated samples were captured to evaluate the coating’s performance.

## 4. Conclusions

In conclusion, we present an investigation into the effect of incorporating different concentrations of GO (1.3, 0.1, and 0.01 wt%) in WPU resins and the impact of functionalizing low-content (0.01 wt%) GO sheets with ethylenediamine (EDA) as an additive to develop anticorrosive coatings for carbon steel surfaces. Potentiodynamic polarization analysis in a 3.5% NaCl solution established a strong correlation between the low GO content in the WPU resin matrix and enhanced anticorrosion properties, particularly for the 0.01 wt% GO-EDA, which achieved high E_corr_ (−117.82 mV) and low i_corr_ (3.70 × 10^−^⁹ A cm^−2^) values and an inhibition corrosion efficiency (η) of 99.60%. Raman imaging mapping analysis demonstrated that increasing the GO content as an additive in the WPU matrix leads to the agglomeration of GO sheets, creating pathways for corrosive species to permeate the WPU resin and reach the carbon steel surface. The sizeable lateral dimensions of the GO sheets, combined with the cross-linking between GO and WPU promoted by EDA, improved the interfacial properties between the GO and WPU polymer matrix. This resulted in better barrier properties, homogeneous dispersion, and a less hydrophilic surface, consequently leading to superior anticorrosion performance of the developed eco-friendly coating.

## Figures and Tables

**Table 1:** Electrochemical parameters obtained from potentiodynamic polarization curves for all studied conditions.

Sample	E_corr_/(V) vs. Ag|AgCl	i_corr_/(A cm^−2^)	Corrosion Rate (mm/Year)	η
WPU	−0.811	9.03 × 10^−7^	1.01 × 10^−2^	-
1.3-GO	−0.379	2.57 × 10^−6^	2.88 × 10^−2^	-
0.1-GO	−0.361	1.02 × 10^−7^	1.15 × 10^−3^	88.70%
0.01-GO	−0.126	9.34 × 10^−9^	1.05 × 10^−4^	99.00%
0.01-GO-EDA	−0.118	3.70 × 10^−9^	4.15 × 10^−5^	99.60%

**Table 2:** Recent works in the literature based on incorporating GO as an additive for developing coatings with anticorrosion properties. Evaluated performance based on corrosion potential (E_corr_) and corrosion current (i_corr_) extrapolated from potentiodynamic polarization curves.

Ref.	Coating	Graphene Derivative	Concentration (wt%)	Application Method	E_corr_ (V)	i_corr_ (A cm^−2^)
[34]	Waterborne hydroxyl acrylic	WHAR MGO	0.50	Bar coater	−0.27	0.90 × 10^−6^
[35]	Epoxy	GO-PANI-PDA	-	Wire bar coater	−0.59	3.83 × 10^−8^
[36]	Waterborne epoxy	CMCS-rGO	0.05	Bar coater	−0.63	3.05 × 10^−10^
[18]	WPU	GO-PNNG	0.05	-	−0.06	4.98 × 10^−10^
[37]	Polyvinyl alcohol	GO-PVA-SiC	10.00	Spray	−0.45	1.22 × 10^−6^
[38]	Epoxy	PA-G-EP	1.00	Bar coater	−0.62	3.10 × 10^−8^
This work	WPU	GO-EDA	0.01	Blade coater	−0.12	3.70 × 10^−9^

WHAR MGO: dispersion of hydroxy acrylic resin with GO modified with 3-aminopropyltriethoxysilane; GO-PANI-PDA: GO modified with polyaniline and polydopamine; CMCS-rGO: rGO functionalized with carboxymethyl chitosan; GO-PNNG: GO modified with aminoethyl aminopropyl isobutyl polyhedral oligomeric silsesquioxane; GO-pva-SiC: GO modified with polyvinyl alcohol silicon carbide nanowires; PA-G-EP: nanocomposite based on epoxy, phytic acid, and graphene. WPU-GO-EDA: GO functionalized with ethylenediamine incorporated into waterborne polyurethane.

## Data Availability

The data presented in this study are available upon request from the corresponding authors.

## Supplementary Materials

The following supporting information can be downloaded at: https://www.mdpi.com/article/10.3390/molecules29174163/s1, Figure S1: UV-vis spectrum of graphene oxide (GO) dispersion. Figure S2: SEM micrographs of the GO sheets samples on Si substrates, obtained in different areas and magnification. (i,ii) 2700×, (iii) 3300×, (iv) 3700×, and (v,vi) 5000×, showing the sizeable lateral size of the GO sheets. Figure S3: SEM image (A), EDS mapping (chemical composition) (B), and EDS spectrum (C) of the corresponding area of the carbon steel sample used in this work. The sample is typical of AISI 1070 carbon steel, containing 7.4% carbon and 90.8% iron. Supplementary Video S1: Real-time Raman imaging mapping of the 0.01-GO sample; Supplementary Video S2: Real-time Raman imaging mapping of the 0.01-GO-EDA sample; Supplementary Video S3: Real-time Raman imaging mapping of the 0.1-GO sample; Supplementary Video S4: Real-time Raman imaging mapping of the 1.3-GO sample.
