# Supplementary material for: Eco-Friendly Waterborne Polyurethane Coating Modified with Ethylenediamine-Functionalized Graphene Oxide for Enhanced Anticorrosion Performance
Source: Molecules. 2024 Sep 3;29(17):4163. doi: 10.3390/molecules29174163 (PMC11397095; doi:10.3390/molecules29174163)
Supplement: Supplementary file 1 [file molecules-29-04163-s001.zip › molecules-3146824-supplementary.pdf]

Supplementary Material

Eco-Friendly Waterborne Polyurethane Coating Modified with Ethylenediamine-Functionalized Graphene Oxide for Enhanced Anticorrosion Performance

Mariel Amparo Fernandez Aramayo [1,2,*], Rafael Ferreira Fernandes [1,2], Matheus Santos Dias [1,2], Stella Bozzo [1,2], David Steinberg [1,2], Marcos Rocha Diniz da Silva [1,2], Camila Marchetti Maroneze [1,2] and Cecilia de Carvalho Castro Silva [1,2,*]

[1] Mackenzie School of Engineering, Mackenzie Presbyterian University, Consolação Street 930, São Paulo 01302-907, Brazil; reomldm@gmail.com (R.F.F.); theeus.santos@gmail.com (M.S.D.); stellabozzo98@gmail.com (S.B.); david.steinberg@mackenzie.br (D.S.); marcosrochadiniz@hotmail.com (M.R.D.d.S.); camila.maroneze@mackenzie.br (C.M.M.)

[2] MackGraphe-Mackenzie Institute for Research in Graphene and Nanotechnologies, Mackenzie Presbyterian University, Consolação Street 930, São Paulo 01302-907, Brazil

* Correspondence: aramayomariel.m@gmail.com (M.A.F.A.); cecilia.silva@mackenzie.br (C.d.C.C.S.)

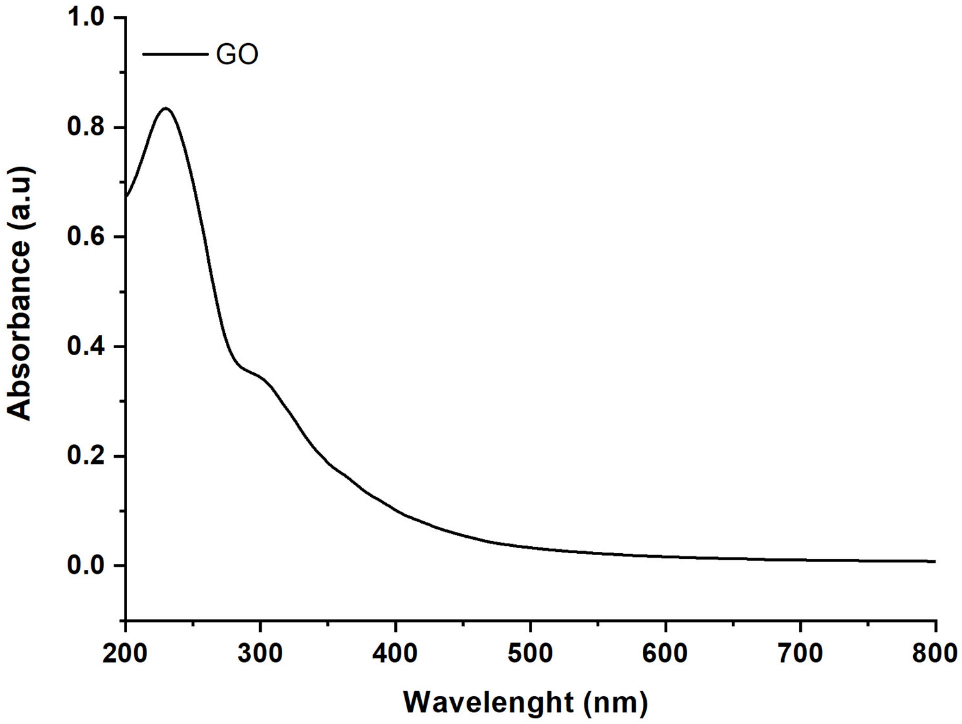

Figure S1. UV-vis spectrum of graphene oxide (GO) dispersion.

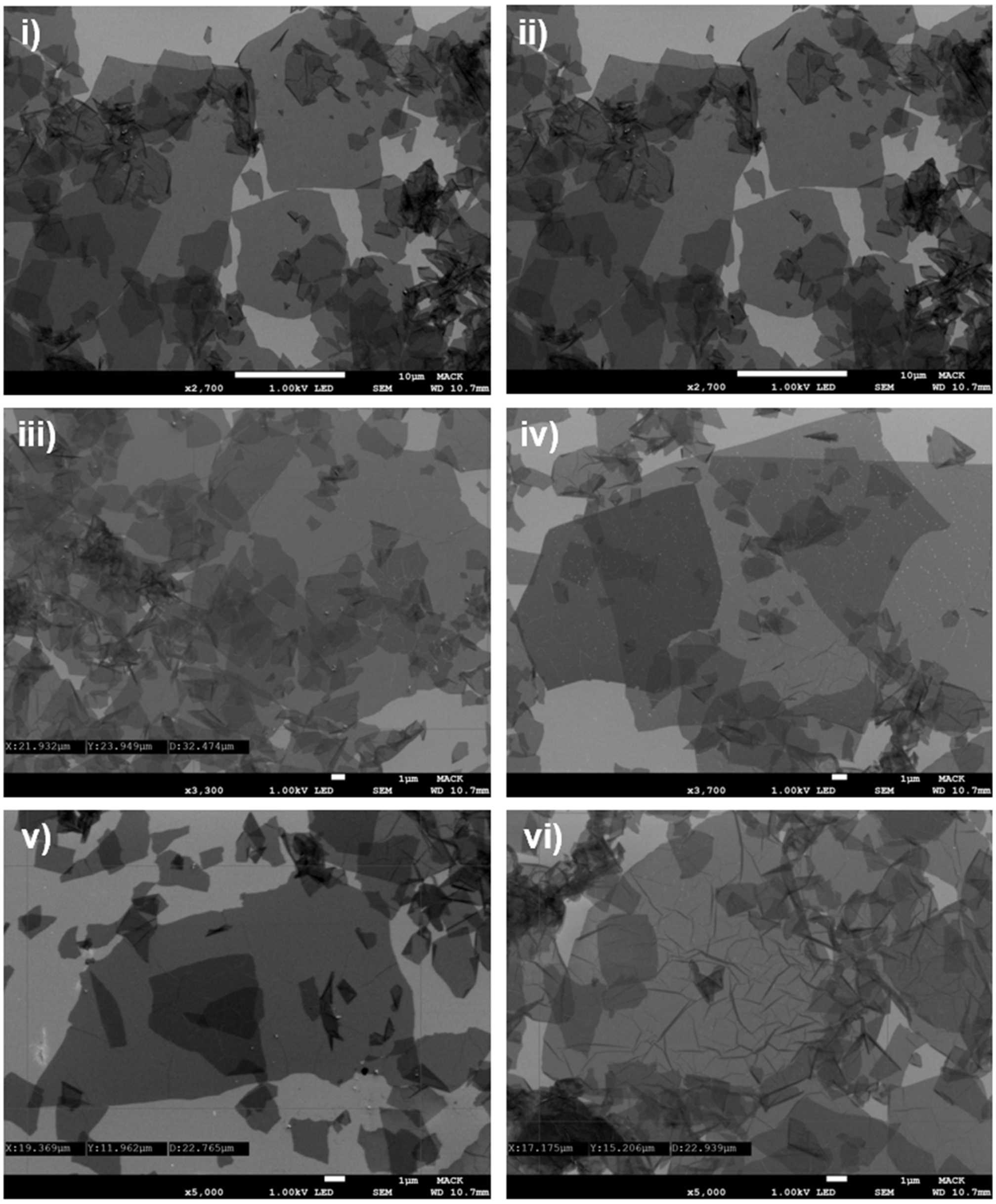

Figure S2. SEM micrographs of the GO sheets samples on Si substrates, obtained in different areas and magnification. i-ii) 2700 x, iii) 3300 x, iv) 3700x and v-vi) 5000 x, showing the sizeable lateral size of the GO sheets.

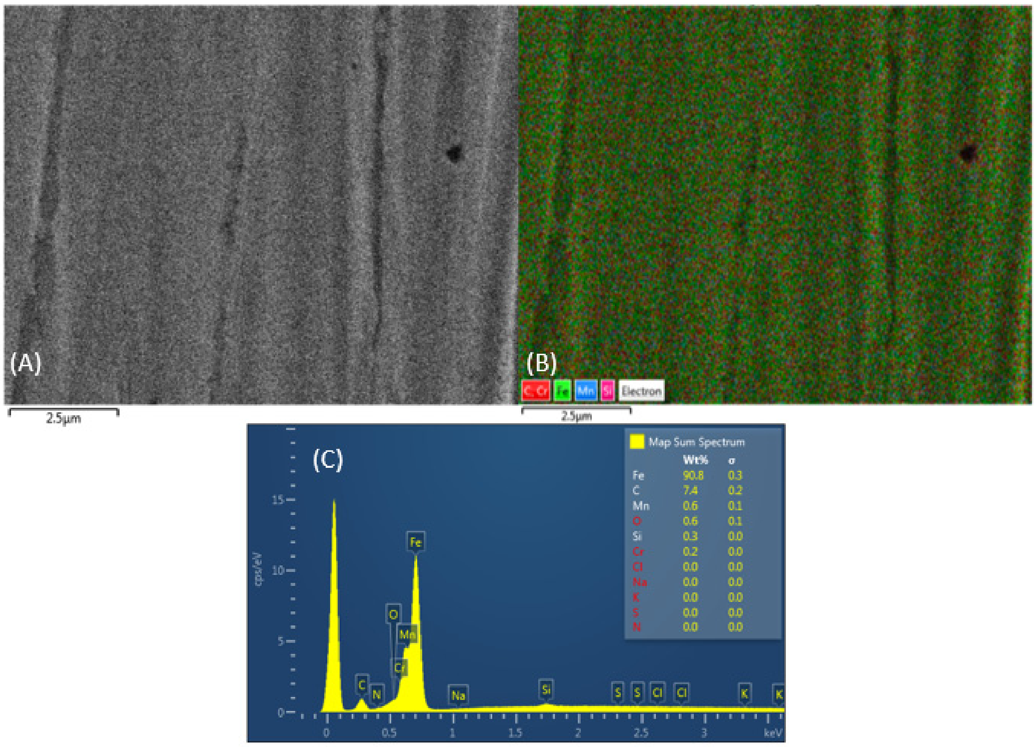

Figure S3: SEM image (A), EDS mapping (chemical composition) (B), and EDS spectrum (C) of the corresponding area of the carbon steel sample used in this work. The sample is typical of AISI 1070 carbon steel, containing 7.4% carbon and 90.8% iron.
